# A 6-month longitudinal and comparative study of corneal biomechanical properties after SMILE with two different optical zone sizes

**DOI:** 10.1371/journal.pone.0337545

**Published:** 2025-12-01

**Authors:** Yizhuo Gong, Xinmeng Wang, Mingkun Yu, Peipei Zu

**Affiliations:** 1 Affiliated Eye Hospital of Shandong University of Traditional Chinese Medicine, Jinan, Shandong, China; 2 Ophthalmology and Optometry Medical School, Shandong University of Traditional Chinese Medicine, Jinan, Shandong, China; 3 Affiliated Hospital of Traditional Chinese Medicine, Binzhou Medical University, Binzhou, Shandong, China; Sanmenxia Central Hospital, Henan University of Science and Technilogy, CHINA

## Abstract

**Purpose:**

To precisely evaluate the independent influence of two different optical zone (OZ) sizes (6.3 mm vs. 6.5 mm) on corneal biomechanical properties within 6 months after Small Incision Lenticule Extraction (SMILE) using multivariable-adjusted statistical models.

**Methods:**

This retrospective study included myopic patients who underwent SMILE between 2022 and 2024. Patients were grouped into two groups based on the planned OZ: Group A (6.3 mm, 44 eyes) and Group B (6.5 mm, 54 eyes). Corneal biomechanical parameters were measured using the Corvis ST preoperatively and at 1, 3, and 6 months postoperatively. Linear Mixed Models (LMM) were used to assess the independent effect of OZ size, adjusting for key baseline covariates and accounting for inter-eye correlation by including a random intercept for patient identifier (ID).

**Results:**

A total of 98 eyes were analyzed. Baseline analysis revealed a significant imbalance between the groups, with Group A (6.3 mm OZ) having higher myopia and a greater corneal stromal ablation depth (both P < 0.001). The central finding from the LMM analysis was that, after adjusting for all covariates, optical zone size (6.3 mm vs. 6.5 mm) had no independent, statistically significant effect on 11 of the 12 biomechanical parameters (all P > 0.05), with the sole exception of Ambrosio Relational Thickness Horizontal (ARTH) (P = 0.012). In contrast, several preoperative covariates, particularly corneal stromal ablation depth and preoperative central corneal thickness, were identified as significant predictors of multiple postoperative biomechanical parameters.

**Conclusion:**

The independent effect of a 0.2 mm difference in optical zone size on corneal biomechanical properties after SMILE appears to be limited. After comprehensive statistical adjustment, the results suggest that preoperative anatomical and surgical parameters, especially corneal stromal ablation depth, are the primary drivers of the postoperative biomechanical response, rather than the minor difference in OZ size itself. This study underscores the importance of confounder adjustment in refractive surgery research and suggests that maximizing the residual stromal bed may be more critical for maintaining corneal biomechanical integrity than fine-tuning the optical zone diameter.

## Introduction

Small Incision Lenticule Extraction (SMILE) has become established as a primary refractive procedure for myopia correction, owing to its demonstrated safety, efficacy, and predictability [1]. Corneal biomechanics, a key indicator reflecting the non-linear elastic and viscoelastic properties of the cornea [2], is influenced by various factors including age [3], sex [4], refractive error [5], and intraocular pressure (IOP) [6]. Within the context of SMILE surgery, the influence of the OZ size on postoperative corneal biomechanics is a topic of significant clinical interest that warrants further investigation, and has garnered increasing attention in recent years. SMILE surgery reshapes the cornea by creating and extracting a lenticule within the corneal stroma. As the stroma accounts for approximately 90% of the total corneal thickness and its regularly arranged collagen fibrils are the primary source of the cornea’s biomechanical strength, any surgical intervention within this layer will alter the mechanical properties of the cornea [7,8].

In keratorefractive surgery, previous studies have established that the choice of optical zone (OZ) size impacts postoperative visual quality [9,10]. However, a larger OZ inevitably involves more extensive removal of corneal stromal tissue, which has raised ongoing concerns about its potential impact on long-term corneal biomechanical stability. Furthermore, the selection of OZ diameter is often correlated with other critical surgical variables, such as the preoperative spherical equivalent and the corresponding ablation depth. Therefore, it is crucial to disentangle whether the observed biomechanical changes are a direct consequence of the OZ size itself, or are confounded by these associated parameters [11]. Furthermore, as the postoperative changes in corneal biomechanics represent a dynamic process [12], and because fully characterizing this timeline requires sufficient longitudinal observation, the patterns of recovery and stabilization over the first several months postoperatively remain incompletely understood. Accurately characterizing these patterns requires a reliable measurement tool.

The Corvis ST is a novel non-contact instrument that provides comprehensive and quantitative assessment of corneal mechanical properties [13]. It is important to clarify that clinical devices like the Corvis ST do not measure the intrinsic material constants of the cornea (e.g., Young’s modulus) in a classic physics sense. Instead, they provide a series of surrogate metrics that reflect the cornea’s overall resistance to deformation, rigidity, and viscoelasticity by documenting the dynamic deformation response of the cornea to a standardized puff of air. Therefore, this study utilized the Corvis ST to conduct a 6-month biomechanical follow-up of patients undergoing SMILE surgery with different OZ sizes. The primary aim was to use multivariable-adjusted statistical models to isolate the independent influence of a minor (0.2 mm) difference in OZ size on postoperative corneal biomechanical characteristics, while accounting for key confounding variables and longitudinal changes, with the aim of providing objective, quantitative biomechanical evidence for personalized surgical planning.

## 1. Subjects and methods

### 1.1 Study design and subjects

This study employed a retrospective, comparative cohort design. Data were retrospectively collected from the medical records of patients with myopia who underwent SMILE surgery at the Refractive Surgery Department of the Eye Hospital of Shandong University of Traditional Chinese Medicine. The records pertained to patients treated from January 1, 2022, to December 30, 2024. For the purpose of this research, the relevant de-identified patient data were accessed by the research team between January 1, 2025, and January 31, 2025. During the initial data extraction from these records, the authors had access to information that could identify individual participants. However, to ensure participant anonymity for the research analysis, all personal identifiers (e.g., names, medical record numbers, exact dates of birth) were removed, and a unique, non-identifiable study code was assigned to each participant before data analysis. Thereafter, the research team only had access to this de-identified dataset for subsequent analysis. All participants had provided written informed consent prior to their surgery, which included consent for their anonymized data to be used for research purposes. All study procedures adhered to the principles of the Declaration of Helsinki and were approved by the Medical Ethics Committee of the Affiliated Eye Hospital of Shandong University of Traditional Chinese Medicine (Approval No.: HEC-KS-2021007KY).

Inclusion criteria were: age ≥ 18 years; spherical error ≥ −10.00 D, cylindrical power ≤ 5.00 D; stable refraction for the past two years (annual change ≤ 0.50 D); IOP < 21 mmHg; cessation of soft contact lens or rigid gas permeable (RGP) lens wear for at least 4 weeks prior to the preoperative examination; preoperative central corneal thickness (CCT) ≥ 480 µm; no history of active eye disease, trauma, or surgery; no keratoconus or suspected keratoconus; good general health and ability to cooperate with examinations and follow-up. Based on the planned surgical OZ diameter, eligible patients were assigned to two groups: Group A (OZ = 6.3 mm) and Group B (OZ = 6.5 mm).

### 1.2 Methods

#### 1.2.1 Preoperative ophthalmic assessment.

All subjects underwent a systematic preoperative ophthalmic examination to assess surgical eligibility and obtain baseline data. Examinations included: (1) visual acuity testing: uncorrected distance visual acuity and corrected distance visual acuity measured using a standard logarithmic visual acuity chart (E-chart); (2) refraction: objective refraction was performed using an auto-refkeratometer (ARK-1, NIDEK, Japan), and the average of three reliable measurements was recorded. This was followed by subjective refraction using a phoropter; (3) IOP measurement: average of three consecutive measurements using a non-contact tonometer (NT-510, NIDEK, Japan); (4) slit-lamp microscopy (SL 800, Carl Zeiss AG, Germany) for assessment of the cornea, anterior chamber, and lens; (5) fundus examination: performed using indirect ophthalmoscopy or fundus photography after pupil dilation with compound tropicamide eye drops(0.5%, from Xingqi Pharmaceuticals); (6) corneal topography and tomography: measurement of corneal morphological parameters using the Pentacam Scheimpflug system (Oculus, Germany); (7) axial length (AL) measurement: AL was measured using the IOLMaster 500 (Carl Zeiss Meditec, Germany), and the average of three reliable measurements was recorded; and (8) baseline corneal biomechanical assessment: Performed using the Corvis ST to obtain preoperative biomechanical parameters, including the biomechanically-corrected intraocular pressure (bIOP) used for baseline analysis.

#### 1.2.2 Corneal biomechanical measurement.

Standardized measurements of corneal biomechanical parameters were performed preoperatively and at 1, 3, and 6 months postoperatively using the Corvis ST (1.6b2507(E-Staging),Oculus, Wetzlar, Germany). During measurement, the patient was seated comfortably with their chin stabilized on the chin rest and forehead against the headrest, maintaining steady fixation on the target.

All measurements were performed by the same experienced technician. To ensure data quality, a single high-quality reading was acquired for each eye at each time point. The validity of each measurement was determined by the device’s built-in Quality Score (QS), and only readings with an “OK” designation were included in the final analysis. If a measurement was flagged with a low QS or showed obvious artifacts (e.g., from blinking or poor alignment), it was immediately discarded, and the patient was allowed to rest briefly before a new measurement was performed until a valid reading was obtained. A total of 12 primary biomechanical parameters were collected for analysis. The abbreviations, full names, and biomechanical significance of these parameters are detailed in Table 1.

**Table 1:** Description and biomechanical significance of corvis ST parameters.

Abbreviation	Parameter	Biomechanical significance
**A1L, A2L**	First/Second Applanation Length	Reflects the deformation range of the cornea during compression and rebound phases.
**A1V, A2V**	First/Second Applanation Velocity	Reflects the dynamic response velocity of the corneal apex during compression and rebound.
**Radius**	Radius of Curvature at Highest Concavity	Indicates the degree of corneal bending at maximum concavity. A smaller radius signifies greater curvature and lower rigidity.
**SP-A1**	Stiffness Parameter at First Applanation	A composite index designed to quantify the overall stiffness of the cornea. A higher value indicates a stiffer cornea.
**SSI**	Stress-Strain Index	A parameter intended to simulate the intrinsic material strength of the corneal tissue. A higher value indicates greater material strength.
**IR**	Integrated Radius	Reflects the extent and duration of corneal indentation. A larger value typically indicates lower corneal stiffness.
**DA**	Deformation Amplitude	The maximum displacement of the corneal apex under the air puff. A higher value indicates the cornea deforms more easily, signifying weaker resistance.
**ARTH**	Ambrosio Relational Thickness Horizontal	A composite index combining corneal thickness and its progression profile, used in screening for ectatic risk.
**PD**	Peak Distance	The distance between the two highest peaks of the cornea at maximum concavity, reflecting the shape of the deformation.
**DA Ratio**	Deformation Amplitude Ratio	The ratio of the deformation at the corneal apex to that at a 2-mm paracentral location. A higher ratio suggests the center is weaker relative to the periphery.

#### 1.2.3 Surgical method.

All surgeries were performed by the same experienced surgeon in a standard laminar flow operating room. Standard preoperative protocols included periorbital skin disinfection and draping. Topical anesthesia was administered using proparacaine hydrochloride eye drops.

The procedure was performed using the VisuMax femtosecond laser system (Carl Zeiss Meditec, Jena, Germany). Patients fixated on the target light while the laser system scanned, sequentially creating the posterior surface, edge cut, and anterior surface of the lenticule. The corneal cap thickness was uniformly set at 120 µm. After laser scanning, the surgeon used a micro-dissector to carefully separate and completely remove the corneal stromal lenticule through a small 2–4 mm incision superiorly. After confirming no residual stromal tissue, the corneal incision and interface were irrigated with balanced salt solution. Standard postoperative topical medication was prescribed, including: (1) One drop of Levofloxacin Hydrochloride Eye Drops (5 ml: 24.4 mg, from Bausch & Lomb) four times daily for 1 week; (2) One drop of Loteprednol Etabonate Ophthalmic Suspension (5 ml: 25 mg, from Bausch & Lomb) three times daily with a weekly taper; and (3) One drop of Sodium Hyaluronate Eye Drops (0.1%, from Wanhan Pharmaceuticals) four times daily for 1 month.

The 6.3 mm and 6.5 mm optical zones were chosen for this study as they represent two of commonly used settings in clinical practice for SMILE surgery. This selection allows for the investigation of whether a minimal, 0.2 mm difference—a frequent decision point for surgeons balancing between enhancing visual quality and maximizing stromal tissue preservation—results in a discernible impact on postoperative corneal biomechanics.

### 1.3 Statistical analysis

All data analysis was performed using SPSS software (version 27.0, IBM Corp., Armonk, NY, USA). The normality of quantitative data was assessed using the Shapiro-Wilk test. Normally distributed data were expressed as mean ± standard deviation (M ± SD).

Initially, independent samples t-tests were used to compare baseline characteristics between the two groups. To evaluate the degree of correlation between data from fellow eyes in subjects where both eyes were included, an Intraclass Correlation Coefficient (ICC) analysis was performed on key biomechanical parameters for this subset of patients.

Given the significant between-group imbalances identified in the baseline analysis (see Results section) and the significant inter-eye correlations revealed by the ICC analysis, Linear Mixed Models (LMM) were ultimately employed for the primary longitudinal analysis. In these models, the absolute values of the biomechanical parameters at each postoperative time point (1, 3, and 6 months) were set as the dependent variables. Group (Group A vs. Group B), Time, and their interaction term were included as fixed effects. To adjust for baseline differences, preoperative age, spherical equivalent (SE), central corneal thickness (CCT), corneal stromal ablation depth, axial length (AL), and biomechanically-corrected intraocular pressure (bIOP) were all included in the models as covariates. To account for the correlation of data from the two eyes of the same individual, Patient ID was included as a random effect (random intercept). All statistical tests were two-tailed, and a *P*-value less than 0.05 was considered statistically significant.

## Results

### Baseline characteristics

A total of 98 eyes were included in the final analysis and allocated into two groups based on the planned optical zone (OZ) diameter: Group A (6.3 mm OZ, n = 44) and Group B (6.5 mm OZ, n = 54). The demographic and preoperative characteristics of the two groups are presented in Table 2. There were no statistically significant differences between the groups in terms of mean age (25.50 ± 5.87 vs. 26.13 ± 7.20 years, P = 0.634), preoperative central corneal thickness (CCT) (548.20 ± 24.53 vs. 554.26 ± 21.78 µm, P = 0.199), axial length (AL) (25.59 ± 0.94 vs. 25.48 ± 0.85 mm, P = 0.557), or biomechanically-corrected intraocular pressure (bIOP) (16.21 ± 1.75 vs. 16.53 ± 2.45 mmHg, P = 0.456). However, a significant baseline imbalance was observed between the two groups for key refractive and surgical parameters. Group A had a significantly higher mean preoperative spherical equivalent (SE) compared to Group B (−5.46 ± 0.93 D vs. −3.98 ± 1.05 D, P < 0.001). Consequently, the mean corneal stromal ablation depth was also significantly greater in Group A than in Group B (106.77 ± 13.67 µm vs. 89.57 ± 13.42 µm, P < 0.001).

**Table 2:** Demographic and preoperative characteristics by optical zone group.

Characteristic	Group A (n = 44)	Group B (n = 54)	t value	P value
**Age (years)**	25.50 ± 5.87	26.13 ± 7.20	−0.477	0.634
**SE (D)**	−5.46 ± 0.93	−3.98 ± 1.05	−7.361	<0.001
**CCT (µm)**	548.20 ± 24.53	554.26 ± 21.78	−1.293	0.199
**Ablation depth (µm)**	106.77 ± 13.67	89.57 ± 13.42	6.245	<0.001
**AL (mm)**	25.59 ± 0.94	25.48 ± 0.85	0.589	0.557
**bIOP (mmHg)**	16.21 ± 1.75	16.53 ± 2.45	−0.749	0.456

*Data are presented as mean ± standard deviation. P-values were derived from independent samples t-tests.*

Abbreviations: *SE, spherical equivalent; D, diopters; CCT, central corneal thickness; Ablation depth, corneal stromal ablation depth; AL, axial length; bIOP, biomechanically-corrected intraocular pressure.*

### Inter-eye correlation analysis

Given that data from both eyes were included for a subset of subjects, we next evaluated the degree of inter-eye correlation for the 6-month postoperative change in all biomechanical parameters. This analysis was performed on the subset of 43 patients for whom data from both eyes were available. The results of the Intraclass Correlation Coefficient (ICC) analysis are detailed in Table 3. The findings revealed moderate to high and statistically significant correlations between fellow eyes for the majority of key biomechanical parameters. Notably, the inter-eye correlation was strongest for the change in deformation amplitude (ΔDA) (ICC = 0.727, P < 0.001), with other critical parameters such as the change in peak distance (ΔPD; ICC = 0.607, P < 0.001) and Stress-Strain Index (ΔSSI; ICC = 0.575, P < 0.001) also demonstrating significant correlation.

**Table 3:** Inter-eye correlation of the 6-month postoperative change in biomechanical parameters.

Biomechanical Parameter	A1L (mm)	A1V (m/s)	A2L (mm)	A2V (m/s)	PD (mm)	Radius (mm)	DA (µm)	DA ratio (%)	ARTH (mm)	IR (mm)	SP-A1 (mm)	SSI (kPa)
**ICC**	−0.327	−0.002	−0.104	0.623	0.607	0.404	0.727	0.534	0.535	0.525	0.355	0.575
**P value**	0.987	0.504	0.746	<0.001	<0.001	0.004	<0.001	<0.001	<0.001	<0.001	0.009	<0.001

*Analysis was based on the subset of 43 patients for whom both eyes were included. The intraclass correlation coefficient (ICC) was calculated using a two-way mixed model with an absolute agreement definition.*

*Abbreviations: Δ, change from preoperative baseline; A1L/A2L, first/second applanation length; A1V/A2V, first/second applanation velocity; PD, peak distance; DA, deformation amplitude; ARTH, Ambrosio Relational Thickness Horizontal; IR, integrated radius; SP-A1, stiffness parameter at first applanation; SSI, Stress-Strain Index.*

This significant inter-eye dependency violates the assumption of independence required by standard statistical tests (e.g., t-tests, repeated measures ANOVA). Therefore, to appropriately account for this correlated data structure and the previously identified baseline imbalances, Linear Mixed Models (LMM) were employed for the primary longitudinal analysis.

### Longitudinal biomechanical analysis: Linear mixed models

To assess the longitudinal impact of optical zone size on postoperative corneal biomechanics while simultaneously accounting for baseline imbalances and inter-eye correlation, Linear Mixed Models (LMMs) were fitted for the absolute postoperative values (at 1, 3, and 6 months) of the 12 primary biomechanical parameters. The summary of the fixed effects tests for all models is presented in Table 4. The detailed parameter estimates for all fixed effects, including beta coefficients and 95% confidence intervals, are provided in S1 Table. The central finding from the analysis was that, after adjusting for all baseline covariates, the main effect of optical zone group was not statistically significant for 11 of the 12 biomechanical parameters (all P > 0.05). The sole exception was the Ambrosio Relational Thickness Horizontal (ARTH), which showed a significant difference between the groups (P = 0.012).

**Table 4:** Summary of fixed effects from linear mixed models for postoperative biomechanical parameters.

Dependent Variable	Group	Time	Group × Time	Age	Ablation Depth	Preop. SE	Preop. CCT	Preop. AL	Preop. IOP
	P Value	P Value	P Value	P Value	P Value	P Value	P Value	P Value	P Value
**A1L (mm)**	0.422	0.772	0.306	0.280	0.391	0.580	0.370	0.464	0.653
**A1V (m/s)**	0.175	0.201	0.441	0.057	0.185	0.546	0.062	<0.001***	0.036*
**A2L (mm)**	0.226	0.737	0.639	0.112	0.067	0.684	0.226	0.418	0.924
**A2V (m/s)**	0.462	<0.001***	0.473	0.630	0.076	0.799	0.152	0.006**	<0.001***
**PD (mm)**	0.899	0.212	0.871	0.648	0.129	0.799	0.009**	0.009**	<0.001***
**Radius (mm)**	0.621	0.957	0.642	0.566	0.001**	0.883	0.565	0.010*	0.003**
**DA (µm)**	0.873	0.017*	0.747	0.374	0.074	0.296	0.205	0.014*	<0.001***
**DA ratio (%)**	0.709	0.754	0.852	0.002**	<0.001***	0.176	<0.001***	0.059	0.061
**ARTH (mm)**	0.012*	0.003**	0.430	0.668	<0.001***	0.802	<0.001***	0.057	0.325
**IR (mm)**	0.127	0.266	0.331	0.010*	<0.001***	0.256	0.011*	0.001**	<0.001***
**SP-A1 (mm)**	0.668	0.002**	0.548	0.195	0.013**	0.357	<0.001***	0.016*	<0.001***
**SSI (kPa)**	0.603	0.251	0.216	0.189	0.002**	0.896	0.842	0.092	<0.001***

*This table summarizes the P-values from 12 independent Linear Mixed Models (LMMs), with each row representing a model for the specified dependent variable. The dependent variables were the absolute postoperative values at 1, 3, and 6 months. All models included Group (6.3 mm vs. 6.5 mm), Time (1, 3, 6 months), and their interaction as fixed effects. Preoperative baseline values for Age, SE, CCT, Ablation Depth, AL, and IOP were included as covariates. A random intercept for Patient ID was included to account for inter-eye correlation. Abbreviations: Preop., preoperative; SE, spherical equivalent; CCT, central corneal thickness; AL, axial length; IOP, biomechanically-corrected intraocular pressure. For other abbreviations, see* Table 3. * P < 0.05; ** P < 0.01; *** P < 0.001.

Regarding the postoperative trajectory, a significant main effect of Time was observed for several parameters (e.g., A2V, DA, ARTH, SP-A1; all P < 0.05), indicating that these metrics continued to undergo significant longitudinal changes between 1 and 6 months postoperatively. However, the Group × Time interaction was not significant for any parameter, suggesting that the pattern of longitudinal change (i.e., the recovery curve) was parallel between the two optical zone groups. In stark contrast to the generally non-significant effect of the optical zone, several preoperative baseline covariates were identified as powerful predictors of postoperative biomechanics. As shown in Table 4, corneal stromal ablation depth, preoperative CCT, AL and IOP were all significantly associated with multiple outcome parameters. This result strongly suggests that these baseline factors, rather than the 0.2 mm difference in optical zone diameter, are the primary drivers of the postoperative corneal biomechanical response.

## Discussion

Optimizing Small Incision Lenticule Extraction (SMILE) surgical parameters to preserve long-term corneal biomechanical stability is a subject of ongoing clinical investigation [14,15]. This study aimed to evaluate the effect of two commonly used optical zone sizes, 6.3 mm and 6.5 mm, on postoperative corneal biomechanics using a longitudinal design and statistical models that accounted for multiple variables. The primary finding of this study was that after adjusting for a series of key preoperative variables—including refractive error, corneal thickness, and ablation depth—and considering the correlation of data from fellow eyes, the 0.2 mm difference in optical zone size did not have an independent, statistically significant effect on the majority (11 out of 12) of the Corvis ST biomechanical parameters assessed.

A noteworthy exception to this general finding was the Ambrosio Relational Thickness Horizontal (ARTH), which showed a significant difference between the groups (P = .012). The ARTH is a composite index that reflects the corneal thickness profile and is used in screening for ectatic risk. This isolated finding may suggest that while the optical zone size has a limited effect on overall corneal deformability or stiffness, it might still induce subtle, measurable changes to the postoperative corneal morphology or thickness distribution. However, this result should be interpreted with caution. Given that multiple outcome variables were tested in this study, the principle of multiple comparisons suggests that a single positive result could be attributable to chance (i.e., a Type I error). Therefore, while this difference in ARTH is statistically significant, its definitive clinical importance requires further validation in future prospective studies with larger sample sizes.

This finding differs from the results of a preliminary, unadjusted analysis. When performing a simple between-group comparison (e.g., a t-test), we initially observed that the 6.5 mm optical zone group had a significantly greater change in deformation amplitude (ΔDA). The disappearance of this significance after statistical adjustment suggests the presence of confounding bias. Our baseline analysis (Table 2) clearly showed that the 6.3 mm optical zone group had a significantly greater mean corneal stromal ablation depth to correct a higher degree of myopia. The results from the Linear Mixed Models (LMM) indicate that the initially observed between-group difference was likely explained or “absorbed” by stronger predictive factors, such as ablation depth. This highlights that without adequate statistical adjustment for potential confounders, interpretations of results can be skewed.

In contrast to the generally non-significant effect of the optical zone, our LMM analysis (Table 4) identified other key factors that influence postoperative corneal biomechanics. Specifically, corneal stromal ablation depth and preoperative central corneal thickness (CCT) were identified as significant predictors for multiple biomechanical parameters (e.g., DA ratio, ARTH, IR, SP-A1, SSI). This finding is highly consistent with biomechanical principles, as the postoperative structural integrity of the cornea is primarily determined by the thickness of the residual stromal bed (RSB) [11,14,16], which is a direct function of preoperative CCT and ablation depth The evidence from this study, therefore, suggests that the amount of tissue removed (ablation depth) may play a more fundamental role in determining the postoperative biomechanical response than the width of the treatment area (optical zone).

Our longitudinal analysis also provides information on the postoperative corneal recovery process. The main effect of “Time” in the LMM was significant for several parameters reflecting both corneal deformability (e.g., DA) and stiffness (e.g., SP-A1), indicating that the cornea undergoes continuous, measurable biomechanical remodeling between 1 and 6 months postoperatively. This dynamic process is consistent with the known timeline of corneal wound healing, which involves keratocyte remodeling and the establishment of a new biomechanical equilibrium [14,17]. Notably, the Group × Time interaction effect was not significant for any parameter, suggesting that while the cornea continues to change, the recovery patterns (i.e., the slopes of change) were parallel for both optical zone groups.

The results of this study may have a certain reference value for clinical practice. They suggest that when planning SMILE surgery, clinicians should focus on factors proven to have a decisive impact on biomechanics, such as preserving an adequate residual stromal bed (RSB). In contrast, for patients with similar refractive and corneal conditions, the choice between a 6.3 mm and 6.5 mm optical zone may have limited differential impact on biomechanical safety. This finding may allow clinicians to place greater emphasis on other factors, such as pupil size and visual quality demands, to achieve more optimal and personalized treatment outcomes.

This study has several limitations. First, its retrospective design may introduce selection bias. Second, although our 6-month follow-up period revealed short- to mid-term trends, longer-term biomechanical evolution requires further investigation, as complete corneal remodeling may take more time [18]. Finally, this study analyzed the planned optical zone (POZ), not the postoperative effective optical zone (EOZ). It is known that postoperative epithelial hyperplasia and remodeling can cause the EOZ to be smaller than the POZ, which may affect both biomechanics and visual quality [19]. Future prospective studies should consider incorporating measurements of the EOZ. Despite these limitations, the statistical approach (LMM) employed in this study was able to simultaneously address common methodological issues such as baseline imbalance and inter-eye correlation, which helps to enhance the internal validity of our conclusions.

In conclusion, this longitudinal analysis using a multivariable-adjusted model indicates that preoperative anatomical and refractive characteristics, particularly corneal ablation depth and thickness, are the primary determinants of the corneal biomechanical response after SMILE. In comparison, the independent, clinically measurable effect of a 0.2 mm difference between a 6.3 mm and 6.5 mm optical zone appears to be limited. This finding underscores the importance of comprehensive confounder adjustment in clinical research and provides new evidence to help optimize personalized surgical planning in SMILE.

## Supporting information

S1 Table Parameter estimates for the fixed effects in linear mixed models for each biomechanical parameter. (DOCX)

S1 Data Excel workbook containing the data used for analysis. (XLSX)

## References

[1] Evereklioglu C, Sener H, Mutlu SN, Gunay Sener AB, Horozoglu F. Top 50 most-cited articles on SMILE surgery between 2010 and 2022: a correlation comparison between conventional bibliometrics and current altmetrics of research impact. Int Ophthalmol. 2023;43(7):2521–32. doi: 10.1007/s10792-023-02652-y 36869979

[2] Wilson A, Marshall J. A review of corneal biomechanics: mechanisms for measurement and the implications for refractive surgery. Indian J Ophthalmol. 2020;68(12):2679–90. doi: 10.4103/ijo.IJO_2146_20 33229643 PMC7856929

[3] Celebi ARC, Kilavuzoglu AE, Altiparmak UE, Cosar Yurteri CB. Age-related change in corneal biomechanical parameters in a healthy Caucasian population. Ophthalmic Epidemiol. 2018;25(1):55–62. doi: 10.1080/09286586.2017.1351997 28891725

[4] Al-Arfaj K, Yassin SA, Al-Dairi W, Al-Shamlan F, Al-Jindan M. Corneal biomechanics in normal Saudi individuals. Saudi J Ophthalmol. 2016;30(3):180–4. doi: 10.1016/j.sjopt.2016.05.001 28210179 PMC5299105

[5] Qiu K, Lu X, Zhang R, Wang G, Zhang M. Corneal biomechanics determination in healthy myopic subjects. J Ophthalmol. 2016;2016:2793516. doi: 10.1155/2016/2793516 27525109 PMC4972914

[6] Brown L, Foulsham W, Pronin S, Tatham AJ. The influence of corneal biomechanical properties on intraocular pressure measurements using a rebound self-tonometer. J Glaucoma. 2018;27(6):511–8. doi: 10.1097/IJG.0000000000000948 29557828

[7] Wilson SE. Bowman’s layer in the cornea- structure and function and regeneration. Exp Eye Res. 2020;195:108033. doi: 10.1016/j.exer.2020.108033 32339517 PMC7283008

[8] Komninou MA, Seiler TG, Enzmann V. Corneal biomechanics and diagnostics: a review. Int Ophthalmol. 2024;44(1):132. doi: 10.1007/s10792-024-03057-1 38478103 PMC10937779

[9] Moshirfar M, Huynh R, Bundogji N, Tukan AN, Sant TM, McCabe SE, et al. Comparison of 6.0 mm versus 6.5 mm Optical Zone on Visual Outcomes after LASIK. J Clin Med. 2021;10(17):3776. doi: 10.3390/jcm10173776 34501222 PMC8432203

[10] Endl MJ, Martinez CE, Klyce SD, McDonald MB, Coorpender SJ, Applegate RA, et al. Effect of larger ablation zone and transition zone on corneal optical aberrations after photorefractive keratectomy. Arch Ophthalmol. 2001;119(8):1159–64. doi: 10.1001/archopht.119.8.1159 11483083

[11] Liang S, Ji S, Liu X, Chen M, Lei Y, Hou J, et al. Applying information gain to explore factors affecting small-incision lenticule extraction: a multicenter retrospective study. Front Med (Lausanne). 2022;9:837092. doi: 10.3389/fmed.2022.837092 35592861 PMC9110865

[12] Ryan DS, Coe CD, Howard RS, Edwards JD, Bower KS. Corneal biomechanics following epi-LASIK. J Refract Surg. 2011;27(6):458–64. doi: 10.3928/1081597X-20110112-01 21243973

[13] Liu M-X, Zhu K-Y, Li D-L, Dong X-X, Liang G, Grzybowski A, et al. Corneal biomechanical characteristics in myopes and emmetropes measured by corvis ST: a meta-analysis. Am J Ophthalmol. 2024;264:154–61. doi: 10.1016/j.ajo.2024.03.024 38556185

[14] Hashemi H, Roberts CJ, Elsheikh A. Corneal biomechanics after SMILE, femtosecond-assisted LASIK, and photorefractive keratectomy: a matched comparison study. Transl Vis Sci Technol. 2023;12(3). 10.1167/tvst.12.3.12 PMC10029763 36928130

[15] Cao K, Liu L, Yu T, Chen F, Bai J, Liu T. Changes in corneal biomechanics during small-incision lenticule extraction (SMILE) and femtosecond-assisted laser in situ keratomileusis (FS-LASIK). Lasers Med Sci. 2020;35(3):599–609. doi: 10.1007/s10103-019-02854-w 31463819

[16] Pniakowska Z, Jurowski P, Wierzbowska J. Clinical evaluation of corneal biomechanics following laser refractive surgery in myopic eyes: a review of the literature. J Clin Med. 2022;12(1):243. doi: 10.3390/jcm12010243 36615041 PMC9821300

[17] Zhang D, Zhang H, Zheng Y, Fu C, Tian L, Zhai C, et al. Time-dependent changes in corneal biomechanics after small incision lenticule extraction: an in vivo study. BMC Ophthalmol. 2025;25(1):438. doi: 10.1186/s12886-025-04260-z 40745645 PMC12312527

[18] Wang J, Cheng X, Chang W, Zhou X, Zhao Y. Long-term refractive outcomes and corneal remodeling after the SMILE surgery in patients with moderate to extra-high myopia. BMC Ophthalmol. 2025;25(1):363. doi: 10.1186/s12886-025-04171-z 40597045 PMC12220274

[19] Wang X, Jiao Y, Liu P, Ye X, Zhou Y, Wang Q, et al. Comparison of dynamic changes in the effective optical zone in low-moderate and high myopia with a novel method and visual outcomes after SMILE surgery: a 6-month clinical study. Lasers Med Sci. 2025;40(1):331. doi: 10.1007/s10103-025-04586-6 40781497

