# Supplementary material for: A 6-month longitudinal and comparative study of corneal biomechanical properties after SMILE with two different optical zone sizes
Source: PLoS One. 2025 Dec 1;20(12):e0337545. doi: 10.1371/journal.pone.0337545 (PMC12668499; doi:10.1371/journal.pone.0337545)
Supplement: S1 Table — (DOCX) [file pone.0337545.s001.docx]

S1 Table. Parameter Estimates for the Fixed Effects in Linear Mixed Models for Each Biomechanical Parameter.

| Dependent Variable | Fixed Effect (Predictor) | Estimate (β) | 95% Confidence Interval | P Value |
| --- | --- | --- | --- | --- |
| A1L (mm) | (Intercept) | 1.367 | (-0.214, 2.948) | 0.089 |
|  | Group (A vs B) | -0.054 | (-0.194, 0.087) | 0.452 |
|  | Time (1M vs 6M) | 0.003 | (-0.101, 0.107) | 0.951 |
|  | Time (3M vs 6M) | -0.030 | (-0.134, 0.074) | 0.568 |
|  | Age | -0.004 | (-0.011, 0.003) | 0.280 |
|  | Preop. AL | 0.021 | (-0.035, 0.076) | 0.464 |
|  | Ablation Depth | -0.002 | (-0.005, 0.002) | 0.391 |
|  | Preop. SE | -0.001 | (-0.003, 0.002) | 0.580 |
|  | Preop. CCT | 0.001 | (-0.001, 0.003) | 0.370 |
|  | Preop. IOP | -0.005 | (-0.027, 0.017) | 0.653 |
| A1V (m/s) | (Intercept) | 0.336 | (0.250, 0.422) | <0.001 |
|  | Group (A vs B) | 0.003 | (-0.004, 0.009) | 0.430 |
|  | Time (1M vs 6M) | 0.001 | (-0.003, 0.005) | 0.668 |
|  | Time (3M vs 6M) | 0.001 | (-0.003, 0.004) | 0.722 |
|  | Age | 0.000 | (-0.000, 0.001) | 0.057 |
|  | Preop. AL | -0.006 | (-0.009, -0.003) | <0.001 |
|  | Ablation Depth | 0.000 | (-0.000, 0.000) | 0.185 |
|  | Preop. SE | 0.000 | (-0.000, 0.000) | 0.546 |
|  | Preop. CCT | 0.000 | (-0.000, 0.000) | 0.062 |
|  | Preop. IOP | -0.001 | (-0.002, -0.000) | 0.036 |
| A2L (mm) | (Intercept) | 2.053 | (-0.141, 4.247) | 0.066 |
|  | Group (A vs B) | 0.040 | (-0.148, 0.228) | 0.677 |
|  | Time (1M vs 6M) | -0.060 | (-0.193, 0.072) | 0.370 |
|  | Time (3M vs 6M) | -0.039 | (-0.171, 0.093) | 0.562 |
|  | Age | -0.008 | (-0.017, 0.002) | 0.112 |
|  | Preop. AL | -0.032 | (-0.109, 0.046) | 0.418 |
|  | Ablation Depth | -0.005 | (-0.010, 0.000) | 0.067 |
|  | Preop. SE | -0.001 | (-0.004, 0.003) | 0.684 |
|  | Preop. CCT | 0.002 | (-0.001, 0.005) | 0.226 |
|  | Preop. IOP | 0.001 | (-0.028, 0.030) | 0.924 |
| A2V (m/s) | (Intercept) | -0.608 | (-0.783, -0.434) | <0.001 |
|  | Group (A vs B) | -0.001 | (-0.015, 0.014) | 0.943 |
|  | Time (1M vs 6M) | 0.013 | (0.003, 0.023) | 0.009 |
|  | Time (3M vs 6M) | 0.004 | (-0.005, 0.014) | 0.382 |
|  | Age | 0.000 | (-0.001, 0.001) | 0.630 |
|  | Preop. AL | 0.009 | (0.003, 0.015) | 0.006 |
|  | Ablation Depth | 0.000 | (-0.001, 0.000) | 0.076 |
|  | Preop. SE | 0.000 | (-0.000, 0.000) | 0.799 |
|  | Preop. CCT | 0.000 | (-0.000, 0.000) | 0.152 |
|  | Preop. IOP | 0.005 | (0.002, 0.007) | <0.001 |
| PD (mm) | (Intercept) | 4.976 | (3.586, 6.365) | <0.001 |
|  | Group (A vs B) | 0.005 | (-0.100, 0.110) | 0.920 |
|  | Time (1M vs 6M) | -0.025 | (-0.076, 0.026) | 0.329 |
|  | Time (3M vs 6M) | -0.006 | (-0.056, 0.045) | 0.829 |
|  | Age | 0.001 | (-0.005, 0.007) | 0.648 |
|  | Preop. AL | 0.066 | (0.017, 0.115) | 0.009 |
|  | Ablation Depth | 0.002 | (-0.001, 0.006) | 0.129 |
|  | Preop. SE | 0.000 | (-0.002, 0.002) | 0.799 |
|  | Preop. CCT | -0.002 | (-0.004, -0.001) | 0.009 |
|  | Preop. IOP | -0.025 | (-0.038, -0.012) | <0.001 |
| Radius (mm) | (Intercept) | 2.383 | (-1.027, 5.793) | 0.168 |
|  | Group (A vs B) | -0.060 | (-0.331, 0.211) | 0.663 |
|  | Time (1M vs 6M) | -0.037 | (-0.198, 0.124) | 0.651 |
|  | Time (3M vs 6M) | 0.010 | (-0.150, 0.170) | 0.904 |
|  | Age | -0.004 | (-0.019, 0.011) | 0.566 |
|  | Preop. AL | 0.159 | (0.038, 0.279) | 0.010 |
|  | Ablation Depth | -0.013 | (-0.021, -0.005) | 0.001 |
|  | Preop. SE | 0.000 | (-0.006, 0.005) | 0.883 |
|  | Preop. CCT | 0.001 | (-0.003, 0.006) | 0.565 |
|  | Preop. IOP | 0.058 | (0.020, 0.096) | 0.003 |
| DA (µm) | (Intercept) | 2.072 | (1.488, 2.657) | <0.001 |
|  | Group (A vs B) | 0.003 | (-0.041, 0.047) | 0.888 |
|  | Time (1M vs 6M) | -0.020 | (-0.042, 0.001) | 0.065 |
|  | Time (3M vs 6M) | -0.004 | (-0.025, 0.017) | 0.719 |
|  | Age | 0.001 | (-0.001, 0.004) | 0.374 |
|  | Preop. AL | -0.026 | (-0.047, -0.005) | 0.014 |
|  | Ablation Depth | 0.001 | (-0.000, 0.003) | 0.074 |
|  | Preop. SE | 0.000 | (-0.001, 0.000) | 0.296 |
|  | Preop. CCT | 0.000 | (-0.001, 0.000) | 0.205 |
|  | Preop. IOP | -0.015 | (-0.021, -0.010) | <0.001 |
| DA Ratio (%) | (Intercept) | 11.016 | (8.544, 13.488) | <0.001 |
|  | Group (A vs B) | 0.011 | (-0.178, 0.199) | 0.911 |
|  | Time (1M vs 6M) | -0.047 | (-0.143, 0.049) | 0.337 |
|  | Time (3M vs 6M) | -0.017 | (-0.112, 0.079) | 0.732 |
|  | Age | 0.017 | (0.007, 0.028) | 0.002 |
|  | Preop. AL | -0.084 | (-0.172, 0.003) | 0.059 |
|  | Ablation Depth | 0.018 | (0.012, 0.024) | <0.001 |
|  | Preop. SE | 0.003 | (-0.001, 0.006) | 0.176 |
|  | Preop. CCT | -0.010 | (-0.014, -0.007) | <0.001 |
|  | Preop. IOP | -0.023 | (-0.046, 0.001) | 0.061 |
| ARTH (mm) | (Intercept) | 68.530 | (-105.232, 242.292) | 0.435 |
|  | Group (A vs B) | -18.528 | (-31.698, -5.358) | 0.006 |
|  | Time (1M vs 6M) | -11.373 | (-17.858, -4.888) | <0.001 |
|  | Time (3M vs 6M) | -7.486 | (-13.925, -1.047) | 0.023 |
|  | Age | 0.163 | (-0.590, 0.917) | 0.668 |
|  | Preop. AL | -5.973 | (-12.131, 0.186) | 0.057 |
|  | Ablation Depth | -1.504 | (-1.906, -1.102) | <0.001 |
|  | Preop. SE | -0.034 | (-0.300, 0.233) | 0.802 |
|  | Preop. CCT | 0.787 | (0.559, 1.015) | <0.001 |
|  | Preop. IOP | 0.806 | (-0.806, 2.419) | 0.325 |
| IR (mm) | (Intercept) | 20.681 | (15.717, 25.644) | <0.001 |
|  | Group (A vs B) | 0.189 | (-0.187, 0.565) | 0.321 |
|  | Time (1M vs 6M) | -0.130 | (-0.314, 0.054) | 0.164 |
|  | Time (3M vs 6M) | -0.146 | (-0.328, 0.037) | 0.117 |
|  | Age | 0.028 | (0.007, 0.050) | 0.010 |
|  | Preop. AL | -0.313 | (-0.489, -0.138) | <0.001 |
|  | Ablation Depth | 0.028 | (0.017, 0.040) | <0.001 |
|  | Preop. SE | 0.004 | (-0.003, 0.012) | 0.256 |
|  | Preop. CCT | -0.008 | (-0.015, -0.002) | 0.011 |
|  | Preop. IOP | -0.100 | (-0.145, -0.054) | <0.001 |
| SP-A1 (mm) | (Intercept) | -173.178 | (-243.213, -103.142) | <0.001 |
|  | Group (A vs B) | 2.848 | (-2.928, 8.625) | 0.332 |
|  | Time (1M vs 6M) | 6.025 | (2.258, 9.792) | 0.002 |
|  | Time (3M vs 6M) | 5.512 | (1.769, 9.254) | 0.004 |
|  | Age | -0.200 | (-0.504, 0.104) | 0.195 |
|  | Preop. AL | 3.052 | (0.582, 5.522) | 0.016 |
|  | Ablation Depth | -0.207 | (-0.369, -0.045) | 0.013 |
|  | Preop. SE | -0.050 | (-0.157, 0.057) | 0.357 |
|  | Preop. CCT | 0.336 | (0.245, 0.428) | <0.001 |
|  | Preop. IOP | 2.208 | (1.353, 3.063) | <0.001 |
| SSI (kPa) | (Intercept) | 0.270 | (-0.477, 1.018) | 0.474 |
|  | Group (A vs B) | -0.016 | (-0.072, 0.040) | 0.574 |
|  | Time (1M vs 6M) | 0.004 | (-0.022, 0.031) | 0.738 |
|  | Time (3M vs 6M) | 0.008 | (-0.018, 0.034) | 0.532 |
|  | Age | 0.002 | (-0.001, 0.005) | 0.189 |
|  | Preop. AL | 0.023 | (-0.004, 0.049) | 0.092 |
|  | Ablation Depth | -0.003 | (-0.005, -0.001) | 0.002 |
|  | Preop. SE | 0.000 | (-0.001, 0.001) | 0.896 |
|  | Preop. CCT | 0.001 | (-0.001, 0.003) | 0.842 |
|  | Preop. IOP | 0.018 | (0.012, 0.025) | <0.001 |

*The table presents the parameter estimates for all fixed effects from 12 independent Linear Mixed Models (LMMs).*

*Model: The dependent variable for each model was the absolute postoperative value of the specified biomechanical parameter. Fixed effects included Group, Time, their interaction, and preoperative covariates (Age, AL, Ablation Depth, SE, CCT, and IOP). Patient ID was included as a random effect.*

*Interpretation (β): The β for Group represents the mean difference of Group A relative to Group B (reference). The β for Time represents the mean difference of the 1M and 3M timepoints relative to 6M (reference).*

*Abbreviations: β, beta coefficient; CI, Confidence Interval; Preop., preoperative; AL, axial length; SE, spherical equivalent; CCT, central corneal thickness; IOP, intraocular pressure.*
